# Radiation-induced changes in gene expression in rectal cancer specimens

**DOI:** 10.1007/s12094-023-03361-9

**Published:** 2024-01-19

**Authors:** Lily Victoria Sarah Hillson, Ashley Kathryn McCulloch, Joanne Edwards, Philip David Dunne, Sean Michael O’Cathail, Campbell Stuart Roxburgh

**Affiliations:** 1 Wolfson Wohl Cancer Research Centre, School of Cancer Sciences, University of Glasgow, Garscube Estate, Switchback Road, Glasgow, G61 1QH UK; 2 The Patrick G Johnston Centre for Cancer Research, Queen’s University Belfast, Belfast, UK; 3 Cancer Research UK Beatson Institute, Glasgow, UK; 4 Academic Unit of Surgery, Glasgow Royal Infirmary, Glasgow, UK

**Keywords:** Rectal, Cancer, Radiotherapy, Transcriptomics, Serial-sampling

## Abstract

**Purpose:**

The standard-of-care for locally advanced rectal cancer is radiotherapy-based neoadjuvant therapy followed by surgical resection. This article reviews the evidence of molecular changes at the transcriptome level induced through radiotherapy in rectal cancer.

**Methods:**

The PubMed search *“(radiation OR radiotherapy) cancer (transcriptome OR “gene expression”) rectal”* was used. The studies taken forward utilised gene-expression data on both pre-treatment and post-treatment rectal adenocarcinoma biospecimens from patients treated with RT-based neoadjuvant strategies.

**Results:**

Twelve publications met the review criteria. There was variation in approaches in terms of design, patient population, cohort size, timing of the post-radiotherapy sampling and method of measuring gene expression. Most of the post-treatment biospecimen retrievals were at resection. The literature indicates a broad upregulation of immune activity through radiotherapy using gene-expression data.

**Conclusion:**

Future studies would benefit from standardised prospective approaches to sampling to enable the inclusion of timepoints relevant to the tumour and immune response.

## Introduction

In 2016–2018, 42,886 people were diagnosed with colorectal cancer in the United Kingdom; in 2017–2019, 16,808 died from the disease [1]. Rectal cancers comprise one-third of all colorectal cancer cases, and within the rectal cancer population, over half of patients will present with non-metastatic locally advanced rectal cancer (LARC) requiring neoadjuvant treatment [1, 2]. LARC is variably defined in Europe and North America but applies to a cohort of patients who exhibit clinical characteristics associated with more advanced local pelvic disease (e.g. cT3 + , cN + , MRI evidence of extramural venous invasion, a threatened or involved circumferential resection margin). Over the last few decades, improvements in the treatment strategy for rectal cancer have achieved better outcomes for patients with LARC. Management of LARC involves neoadjuvant long-course (LCRT) or short-course (SCRT) radiotherapy-based regimes with or without concurrent or subsequent chemotherapy followed by surgery, applying the principles of total mesorectal excision. LCRT for LARC consists of 45.0 – 50.4 Gy of pelvic radiation in 1.8 – 2 Gy fractions over 5 – 5.5 weeks, usually concurrently with a fluoropyrimidine radio-sensitising agent. SCRT for LARC involves 25 Gy in 5 fractions, followed by chemotherapy with fluoropyrimidine and oxaliplatin. Surgical excision typically occurs 6 – 12 weeks after the completion of neoadjuvant therapy. Neoadjuvant radiotherapy can reduce tumour size, enhance the likelihood of curative surgical excision with clear margins, and reduce local recurrence compared to surgery alone [3]. However, there is a high degree of heterogeneity in tumour response to radiotherapy-based neoadjuvant regimes. Approximately 15% of patients develop a complete response (CR), so most patients have residual disease with varying degrees of response [4, 5]. A poor neoadjuvant response is associated with typically higher relapse following surgery and worse mortality rates [6]. Identifying a pre-treatment biomarker capable of predicting treatment response has long been endorsed as an opportunity to stratify patients into the most appropriate treatment regime, allocating treatment to radiosensitive tumours and avoiding overtreatment in radio-resistant tumours. Several studies have analysed gene-expression data on rectal cancer pre-treatment biopsies or post-treatment resections, employing various technologies to heterogenous clinical cohorts. These studies have failed to identify a reproducible gene profile associated with radiation sensitivity that can be applied in a clinical setting [7–10].

Knowledge of the molecular and cellular changes induced by radiotherapy as the treatment response evolves and how these differ between responder and non-responder groups is pertinent to understanding mechanisms of response and resistance. Details of response and resistance mechanisms could be therapeutically exploited to enhance tumour and immunological responses in patients predicted as poor responders. Radiotherapy delivers ionising radiation, resulting in DNA damage and tumour cell death, which has been shown to induce immunological effects. Immunohistochemical findings demonstrate separately that long-course (50.4 Gy, 1.8 Gy/fraction with concurrent 5-FU; 225 mg/m^2^/day) and short-course (20 Gy, 4 Gy/fraction with concurrent UFT; 400 mg/day) chemoradiotherapy increases the abundance of CD8 T-lymphocytes in rectal tumour tissue 4–6 weeks after the end of radiotherapy, although did not find evidence of a change 1-week after the end of a short-course radiotherapy (25 Gy, 5 Gy/fraction[11, 12]). Furthermore, the expression of immune-checkpoint molecules PD-1 and LAG-3 on immune cells increased after treatment in a cohort of combined SCRT and LCRT cohort (25 Gy, 5 Gy/fraction, 45–50.4 Gy in 25–28 fractions + Capecitabine (825 mg/m^2^[13]). This observation forms the rationale for ongoing attempts to combine immune checkpoint inhibition with radiation to enhance anti-cancer T-lymphocyte cell responses in rectal cancer trials [14]. One method of investigating response mechanisms to radiotherapy is using gene-expression changes to infer the upregulation of biological processes. The advantage of applying a whole transcriptomic approach is that the approach is less biased by the researcher’s hypothesis, which is inherent where there is a smaller panel of selected targets for assessment, such as with immunohistochemistry. Using matched irradiated and non-irradiated cohorts or serial tumour tissue for sampling provides an opportunity to uncover biological processes associated with radiation response and resistance.

This review will explore the current published literature for evidence of molecular changes at the transcriptome level induced through radiotherapy in rectal cancer. In particular, this review aims to appraise previous studies where sampling has been performed at baseline and during or after neoadjuvant therapy.

## Methodology

**Fig. 1:**
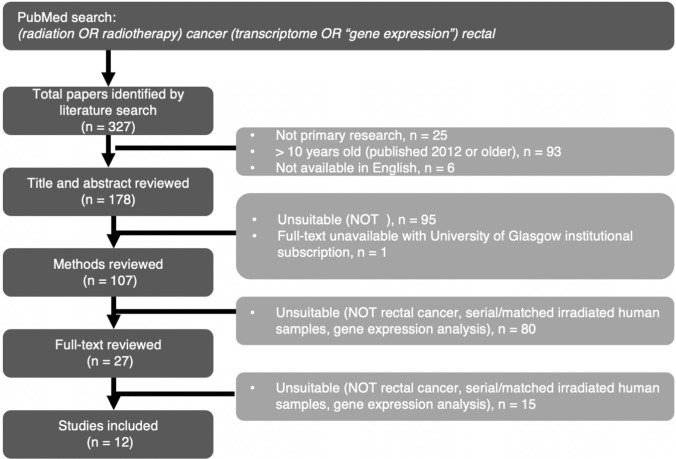
Consort diagram summarising the process of reviewing literature

We sought to identify research articles that used transcriptomic approaches to compare gene and pathway expression in rectal cancer tissue before and after neoadjuvant radiotherapy. We wanted to identify primary research articles written in English and published in the past ten years. PRISMA-P guidelines were consulted before designing the review protocol. The PubMed search term *“(radiation OR radiotherapy) cancer (transcriptome OR “gene expression”) rectal”* was initially used to retrieve *n* = 327 publications. A consort diagram of the review process can be found in Fig. 1. Publications were removed prior to abstract screening because they were not written in English (n = 6), not primary research articles (*n* = 25) or published outside of 01/01/2013 – 31/12/2022. Abstracts and titles from 178 articles were screened to remove articles (*n* = 95) that were explicitly irrelevant (e.g. not rectal cancer, specified as an immunohistochemical study, pre-treatment or resection only specified). One article was removed because the full publication was not accessible using University of Glasgow institutional subscriptions, leaving *n* = 107 to be reviewed for appropriate methodology. Twenty-seven articles used cohorts of patients with irradiated and non-irradiated rectal cancer samples and forms of gene-expression analysis. The full-text review found *n* = 12 articles where gene-expression analysis methods had been used to compare irradiated and non-irradiated samples. Two researchers separately reviewed articles (LH and AM).

## Results

### Description of studies

**Fig. 2:**
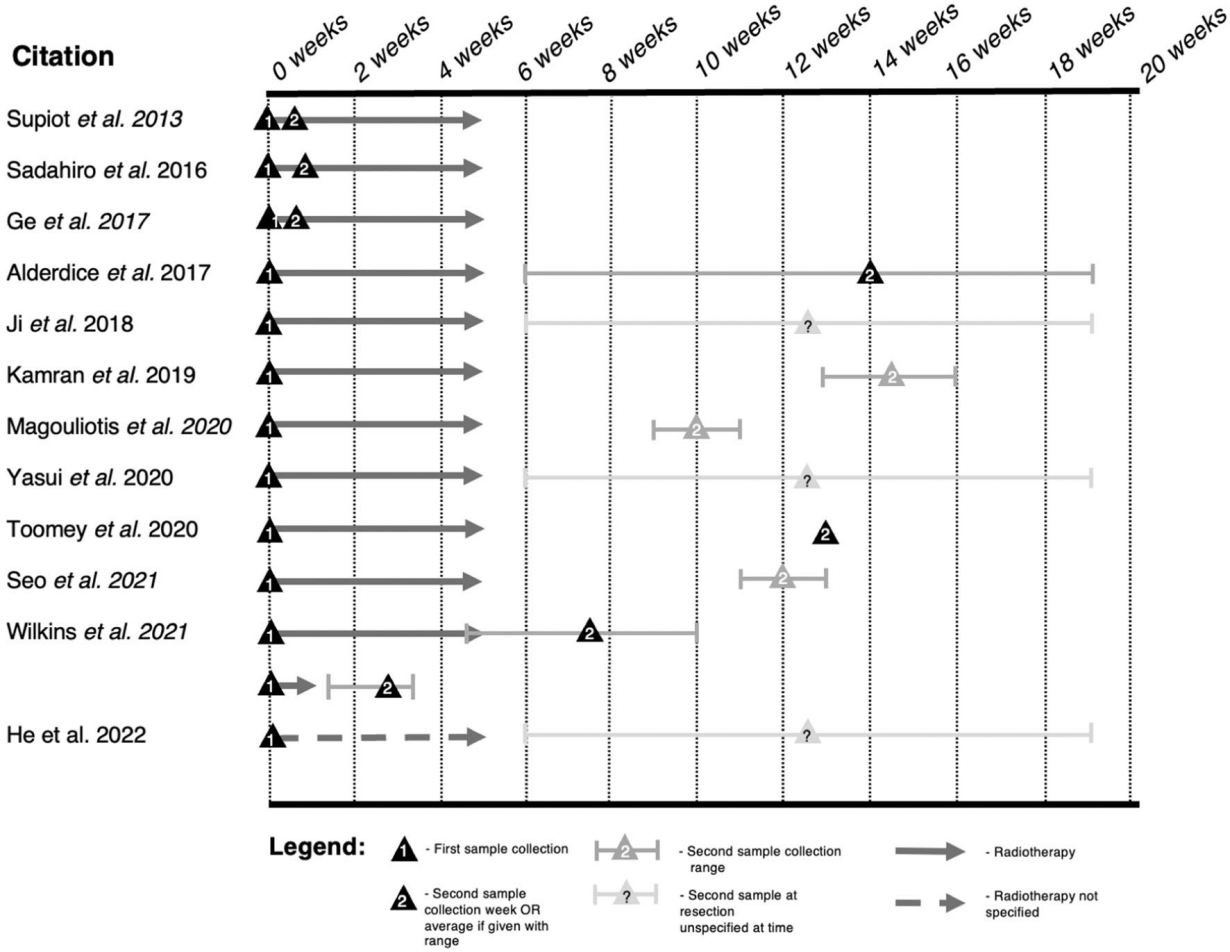
Summary of time points used in publications that look at gene-expression changes in rectal cancer between baseline and during or after neoadjuvant chemoradiotherapy

The search criteria in our methodology retrieved 327 publications. Many papers were unsuitable and attempted to identify a biomarker within pre-treatment samples that reliably predicts clinical response to neoadjuvant-radiotherapy-based strategies. This body of literature has previously been reviewed and will not be further discussed here [7, 9, 10, 15, 16]. Instead, this review will focus on 12 papers identifying gene-expression changes induced by neoadjuvant-radiotherapy-based strategies in rectal cancer using irradiated and non-irradiated samples. Tables 1 and 2 and Fig. 2 provide an overview of these 12 papers. The identified studies were relatively small, with a median cohort size of twenty-one (range 4–82). Only three studies conducted a prospective tissue collection, whilst nine retrospectively assessed archival tissue or published datasets. Five cohorts were strictly defined by clinical TMN stage II or III disease [17] or T3/4 and or node-positive [18–22], which are equivalent. However, other studies had less specific criteria such as ‘operable rectal adenocarcinoma’ [23] or simply ‘locally advanced rectal cancer’ [24–26]. These broader criteria included patients from stage IIA to stage IVA disease [27], T stage 1—4 and N stage 0–2 [24, 25]. The majority of studies had distinct long-course cohorts (n = 10).

**Table 1:** Summary of publications that look at gene expression changes in rectal cancer between baseline and during or after neoadjuvant chemoradiotherapy

Citation	Year	Cohort size	Neoadjuvant treatment	2nd specimen retrieval	Method for RNA analysis	Response measured	Matched
Supiot et al*.* [21]	2013	*n* = 6	45 Gy/25 over 5 weeks	After 4th fraction	Microarray	No	Yes
Sadahiro et al*.* [17]	2016	*n* = 82	45 Gy/25 + a fluoropyrimidine-based regime + (some)bevacizumab	4–9 days after start of CRT	RT-qPCR	Yes—TRG and Japanese Classification of Colorectal Carcinoma (JCCC)	Yes
Ge et al*.* [22]	2017	*n* = 6	45 Gy/25 over 5 weeks	After 4th fraction	Microarray	No	Yes
Alderdice et al*.* [24]	2017	*n* = 40	45 Gy/25 + 5-FU or capecitabine over 5 weeks	Surgical resection at 1–14 weeks post CRT (mean 9-weeks)	Microarray + RT-qPCR	Yes—TRG	Yes
Ji et al*.* [28]	2018	*n* = 22	50 Gy/25 + capecitabine over 5 weeks	Not specified	Microarray	No	No
Kamran et al*.* [18]	2019	*n* = 17	50.4 Gy + 5-FU or capecitabine	Surgical resection at 8–11 weeks post CRT	RNA-sequencing	Yes—pathological complete response	Yes
Magouliotis et al*.* [23]	2020	*n* = 21	None OR 50 Gy/25 + Capecitabine daily over 5 weeks	Surgical resection 4 – 6 weeks post CRT	Microarray	No	No
Yasui et al*.* [27]	2020	*n* = 19	50.4 – 66 Gy + FOLFIRI or capecitabine	Surgical resection	Microarray and RT-qPCR	Yes—tumour regression grade	No
Toomey et al*.* [19]	2020	*n* = 4	50.4 Gy/28 + 5-FU or capecitabine	Surgical resection at 8-week	RNA-sequencing	Yes—pathological complete response	Yes
Seo et al*.* [20]	2021	*n* = 11	50.4 Gy/25 + 5-FU over 5 weeks	6 – 8 weeks after CRT	RNA-sequencing	No	Yes
Wilkins et al*.* [25]	2020	n = 27	25 Gy/5 (SCRT) and 45 Gy/25 (LCRT) + 5-FU	2 – 17 days from start of SCRT. 32 – 78 days from start of LCRT	NanoString panCancer immune panel	Yes—change in tumour cell density	Yes
He et al*.* [26]	2022	*n* = 27	Unknown	Resection	RNA-sequencing	Yes—pathological complete response	No

**Table 2:** Summary of publications that look at gene-expression changes in rectal cancer between baseline and during or after neoadjuvant chemoradiotherapy

Citation	Year	Prospective /retrospective	Multiple biopsies?	Location	Patient disease stage	Method of staging
Supiot et al*.* [21]	2013	Prospective	Yes – 6 from each tumour	France	T > T2, N0/Nx / M0	Unknown
Sadahiro et al*.* [17]	2016	Prospective	Yes – 6 from each tumour	Japan	Stage II or III according to TNM classification	Combination of digital, colonoscopy, CT, transrectal ultrasonography, MRI
Ge et al*.* [22]	2017	Retrospective	Yes – 6 from each tumour	France	T > T2, N0/Nx / M0	Unknown
Alderdice et al*.* [24]	2017	Retrospective	Unknown	UK	‘LARC’ but not defined. tumour status of 1—4 and node status 0—2	Histologically proven locally advanced rectal cancer
Ji et al*.* [28]	2018	Retrospective	Unknown	China	Not specified. TNM I—IV	Endorectal ultrasonography or pelvic MRI or CT
Kamran et al*.* [18]	2019	Retrospective	Unknown	US	T3/4 and/or node-positive	Histologically proven locally advanced rectal cancer
Magouliotis et al*.* [23]	2020	Retrospective	N/A	Norway	Operable rectal adenocarcinoma	Unknown
Yasui et al*.* [27]	2020	Prospective	N/A	Japan	Clinical stage IIA – IVA	Unknown
Toomey et al*.* [19]	2020	Retrospective	Unknown	US / Ireland	T3/4 and/or node-positive	Pathological AJCC or TNM
Seo et al*.* [20]	2021	Retrospective	Unknown	South Korea	T3/4 and/or node-positive	Pelvic MRI
Wilkins et al*.* [25]	2021	Retrospective	Unknown	United Kingdom	‘LARC’ but not defined. tumour status of 1—4 and node status 0—2	AJCC TRG
He et al*.* [26]	2022	Retrospective	Unknown	China	Locally advanced rectal cancer	Unknown

Eight papers compared matched pre-treatment and post-treatment samples within the same patient cohort, whilst four used irradiated and non-irradiated samples from different patients. There was significant variation in the timing of the irradiated biopsy across the studies (Fig. 2). Two studies, working from the same dataset, reported on biopsies after just three fractions of radiotherapy [21, 22]. Sadahiro et al. used biopsies taken one week after starting a LCRT regime [17]. Three studies compared pre-treatment biopsies to post-nCRT resected samples at either 6–8 weeks [20], 8—weeks [19] and 8–11 weeks after finishing radiotherapy [18]. The study, which combined SCRT and LCRT regimes, had a median post-treatment sampling of 11 (range 2–17) and 53 days (range 32–78 respectively). Three papers did not specify the time point for the post-treatment samples [26, 28], but as they were taken from surgical specimens, it is assumed to be between 6 and 12 weeks after neoadjuvant therapy.

Within this literature, various approaches to measuring gene expression have been used. Three studies measured the expression of a targeted selection of genes through the use of reverse transcriptase quantitative polymerase chain reaction (RT-qPCR), whilst ten evaluated thousands of candidates in a transcriptomic approach by microarray (*n* = 6) or RNA-sequencing (*n* = 4) and one used NanoString nCounter. Bias is inherent in targeted approaches; transcriptomic approaches are significantly more helpful in a field with minimal existing knowledge. A trend can be observed whereby earlier studies typically have larger sample sizes and use RT-qPCR to look at a small number of features. More recent studies with smaller sample sizes used more modern technologies to compare thousands of features through transcriptomic approaches such as microarray and RNA-sequencing (Table 1).

### Evidence for immune activation through radiotherapy

Utilising gene-expression data to characterise an immune response to LCRT is a theme in many of the papers in this literature; however, there is no publication focussing on immune changes following SCRT. Notably, all the immune modifications to be described were found by comparing pre-treatment with post-treatment resected samples taken 6–11 weeks after neoadjuvant therapy. Evidence of immune response upregulation was seen in resected specimens eight weeks after LCRT [20, 28]. More specifically, Ji et al*.* used Reactome pathway analysis to show upregulation of interferon signalling, antigen presentation, class I MHC-mediated antigen processing and presentation, peptide-ligand binding receptors co-stimulation from the CD28 pathway in irradiated compared to non-irradiated samples at the time of resection [28]. Similarly, Seo et al*.* reported upregulation of signatures of interferon-$\gamma$, cytolytic activity and general immune activation using their n = 11 cohort and publically available data set GSE15781 [20]. Generally, immune activation was observed in tumours during and after nCRT; however, co-upregulation of PD-1 signalling suggests an interplay with negative regulators of the adaptive immune response, such as checkpoint inhibition [28]. In addition, Yasui et al. reported the upregulation of immune-suppressive cytokines (*IL-6* and *IL-10*) and immune checkpoint genes (*B7-H3* and *B7-H5*) [27].

In addition to identifying immune activation and processes, some sought to characterise the differential immune infiltrate. Within the innate immune system, activated dendritic cells, monocytes, M0 macrophages, M1 macrophages, M2 macrophages, natural killer cells, plasma cells and resting mast cells were upregulated following radiotherapy [18–20, 24, 25, 28]. Conversely, activated mast cells and resting dendritic cells were reduced following radiotherapy [18, 20]. There was disagreement regarding neutrophils, with Ji et al*.* finding that radiotherapy decreased neutrophils whilst Seo et al. reported increased neutrophils in post-treatment samples [20, 28]. Both cohorts of patients received LCRT radiotherapy, but only Seo et al*.* specified a time point for the post-treatment sample. One possible explanation for the discrepancy is that infiltration of neutrophils occurs temporally. In addition, cytotoxic CD8 T-cells, helper CD4 T-cells and naïve B cells increased in the adaptive immune compartment, whilst naïve CD4 T-cells and B-Memory cells decreased post-radiotherapy [18, 20]. Another source of discrepancy or false results is the lack of specificity for RNA markers of immune lineages. Validation of bulk transcriptomic immune signatures showed no significant difference in CD4 and CD8 T lymphocyte populations before and after nCRT; however, CD4 tended to decrease whilst CD8 tended to increase [18]. Validation in a different study agreed that there was no significant difference in CD8 T-cells after radiotherapy but CD4 T-cells tended to decrease whilst CD204 macrophages and FOXP3 + T-regulatory cells increased [27]. However, other studies did not seek validation of changes in immune populations by immunohistochemistry [20, 26, 28].

### Other molecular changes and cellular processes through radiotherapy

Seo et al*.* evaluated gene set enrichment beyond the immune system to identify upregulated cellular processes following neoadjuvant therapy; they reported enrichment of cell adhesion and extracellular matrix organisation within the tumour 6 – 8 weeks after radiotherapy [20]. Conversely, there was a negative enrichment for cell-cycle-related terms and KEGG ‘Mismatch Repair’ post-nCRT [20]. Another study which identified 690 differentially regulated genes used the online search tool DAVID to report enrichment for ‘positive regulation of transport’ and ‘cardiac muscle contraction’ among the upregulated genes and ‘cell migration’, ‘extracellular matrix organisation’ and ‘blood vessel development’ within the downregulated genes [22]. Supiot et al. reported that genes involved in protein metabolism, ion transport, transcription, signal transduction, cell adhesion, immune response and apoptosis increased after radiotherapy [21]. Further to Ge et al. and Supiot et al. reporting an enrichment for transport-related processes, Magouliotis et al. reported that *AQP3* expression was decreased following radiation [23]. Yasui et al. reported on the differential expression of epithelial to mesenchymal transition-associated genes in irradiated and non-irradiated tissues [27]. In small studies, there were insufficient differentially regulated genes to apply signatures by pairwise geneset enrichment or overrepresentation analysis [21, 27]. Studies did not provide validation of transcriptomic findings [20–22, 27]. Two independent studies showed increased gene expression of a regulator pyrimidine synthesis thymidine phosphorylase after nCRT(*TYMP;* [17, 29]).

### Molecular and immunological changes associated with response and resistance to radiotherapy

Wilkins et al. reported that an Inflamed T-cell gene-expression profile was significantly increased in the good responding group, defined by a significant reduction in tumour cell density, but not in the poor responding group (no change or increase in tumour cell density) [25]. Furthermore, they noted that the network of top-upregulated MSigDB Hallmarks: ‘Interferon $\gamma$ response’, ‘Allograft rejection’, ‘IL-6-JAK-STAT3 signalling’, ‘Inflammatory response’ and ‘TNF $\alpha$ signalling via NF $\kappa$ B’ after radiotherapy in good responders was suggestive of a viral-like response [25]. Ingenuity pathway analysis revealed that ‘Natural Killer Cell Signalling’ and ‘Crosstalk between Dendritic Cells’ were uniquely enriched in Tumour Regression Grade (TRG) three resections compared to TRG 1, a finding supported by differential NK cell MCP-counter estimates and natural killer cell marker *NCR1* expression between TRG1 and TRG3 tumour resection [24]. Although not statistically significant, the abundance of NK cells by MCP-counter estimate tended to increase in TRG3 tumours, whilst TRG1 patients showed a mixture of increases and decreases. Alderdice et al. provided validation of higher NK cells post-treatment in TRG3 tumours by CD56 immunohistochemistry. On the single gene level, there was a significant negative correlation between Day7/Pre-treatment ratios of *HIF1A, DPYD* and *TYMP* gene expression, and TRG [17]. Whilst He et al. focussed on identifying immune changes in line with their immune prognostic signature, they could not find significant changes in the small cohort [26]. Generally, the literature points to differential immune activity between responders and non-responders but provides little detail on which molecular and immune changes are associated with favourable responses.

### Differential immunological response between short-course and long-course radiotherapy?

No studies have reported on the gene-expression-based immune changes post-short-course radiotherapy. It is known that the fractionation of radiotherapy influences the immunological response to treatment [30]. Improving the understanding of this in patients is relevant to the design of trials combining immune-oncology agents with radiotherapy.

## Discussion

This review has summarised the small body of literature that utilises serial samples taken at baseline and during or after a course of radiotherapy-based on neoadjuvant strategies to measure changes in gene expression. There is significant variation in the study approach in terms of design, defining the patient population, cohort size, timing of the post-radiotherapy sampling and method of measuring gene expression. Here we will briefly discuss the significance of some of these variables for successfully identifying biologically relevant changes.

The patient cohorts in this literature are typically small, particularly those utilising transcriptomic approaches. The limited size of these cohorts is particularly pertinent due to the inherent heterogeneity of biopsied tumour samples. Intratumoral heterogeneity dictates that the transcriptomic profile of a small tumour biopsy may not be representative of the whole tumour [31]. The issue of heterogeneity is confounded in retrospectively organised studies where investigators rely on single diagnostic biopsies. Two prospective studies recognised the opportunity to improve this and retrieved six pre-treatment biopsies per patient [17, 21]. However, the literature has poorly addressed intertumoral heterogeneity during the analysis by not attempting to stratify patients by consensus molecular subtype classification or treatment outcome. A larger sample size is needed to identify significant biological changes to counteract the noise created by heterogeneity.

Most studies reviewed here included LARC patients according to the most popularised definition of LARC (stage II or III). However, the cohorts were more broadly defined elsewhere, resulting in the inclusion of earlier and later-stage tumours. The inconsistent definition of patient cohorts with rectal adenocarcinoma in this literature reflects the different definitions of LARC globally. European Society for Medical Oncology defines LARC as stage III clinical TNM alone [32]; this may reflect the looser definition of LARC used in the US, encompassing stage II and III disease [33]. Inconsistency between defining LARC in clinical studies and clinical practice limits the applicability of study findings to real patient populations. Furthermore, only one study stated that all tumour staging had been confirmed by magnetic resonance imaging [20].

A strength of the literature to date is the profiling of cohorts treated to a standard-of-care neoadjuvant treatment regime. Most received LCRT, so they have the potential to inform on mechanisms of tumour response currently seen in LCRT patients in the clinic. However, short-course regimes are poorly represented in this literature. Since short-course regimens are increasingly used in the clinic, with neoadjuvant chemotherapy, and may well become the standard-of-care, future studies should not overlook them [34]. Nevertheless, it will be helpful to include and stratify all available standard-of-care treatment options in future studies to assess their differing effects on tumour biology. Such comparison may inform which patients will respond to which regime or which is best suited to combination with immunotherapy.

There is significant variation in the timing of the post-treatment sampling. Since the precise timing of tumour and immune response to radiotherapy regimes is poorly defined, hence the necessity for these studies, it is difficult to judge the appropriate timing of post-treatment sampling. Regardless, many timepoints have emerged through convenience rather than a specific scientific rationale. Sampling after just three fractions [21, 22] or up to a week after nCRT [17] may be inappropriate to capture mechanisms of response and resistance as they evolve in the 6–18 week window between the beginning of nCRT and surgery. On the other end of the spectrum, four studies compared pre-treatment biopsies with resected samples 6—11 weeks after radiotherapy. Tumour regression begins during radiotherapy and continues in the weeks following; therefore, 6—weeks after completing radiotherapy, the fate-determining window where mechanisms of response and resistance occur will have passed. In patients with CRs, the post-radiotherapy samples will be tumour-free, thus making gene-expression analysis of the post-treatment responding tumour impossible. Studies approached this issue differently; Kamran et al. included CRs, presumably measuring gene expression where the tumour once was, potentially capturing residual immune activity [18]. Conversely, Toomey et al*.* excluded patients with CRs, thus potentially ignoring signatures associated with the strongest anti-tumour responses [19]. Regardless, neither approach can identify gene-expression changes in a tumour undergoing a significant response to radiotherapy. The publicly available dataset GSE15781 used by Magouliotis et al*.* and Seo et al*.* includes microarray gene-expression data from patients who underwent resection 4–6 weeks after finishing radiotherapy and patients who underwent resection without neoadjuvant radiation [20, 23]. These two patient cohorts emerged because some patients were judged unfit for neoadjuvant radiotherapy due to their condition [35]. Thus, these samples cannot provide a valid comparison of irradiated and non-irradiated tissues when differences observed may be confounded by the patient’s condition resulting in them being allocated to a particular treatment arm. The timing of the post-treatment biopsy is critical; therefore, failure to specify a timepoint can limit the interpretation of the findings [24, 27, 28]. Here we have identified a significant need for prospectively organised future studies to identify changes in gene expression between baseline and 1–12 weeks after starting radiotherapy to identify biological and immune changes associated with tumour response and resistance.

So far in the discussion, we have highlighted sources of heterogeneity and unreliability within this body of literature. Given this, the interpretation of these studies must be cautious. The body of literature broadly suggests that transcriptomic approaches to identify gene-expression changes through radiotherapy generally point to increased immune activity after radiotherapy. However, many immune signatures can be co-correlated, and immunohistochemistry validation of immune cell infiltration did not show a clear change in lymphocyte populations. This highlights the need for gene expression reporting to be accompanied by appropriate orthogonal protein or cell-level validation methods such as immunohistochemistry or flow cytometry.

## Conclusion

**Fig. 3:**
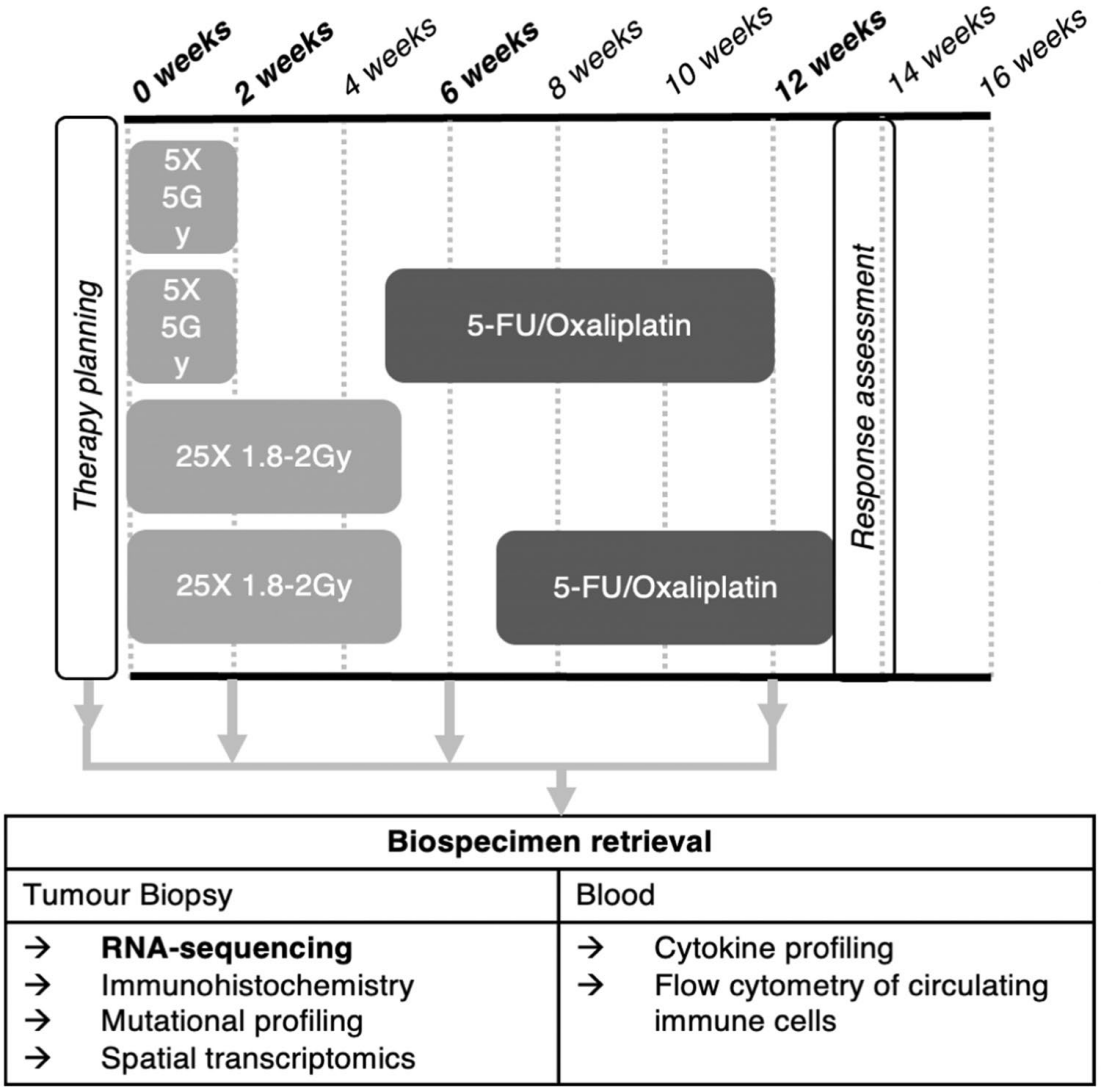
Schematic summary of our current prospective clinical study involving short-course and long-course treated rectal cancer patients with sampling at baseline, 2-week, 6-week and 12-week following the start of radiotherapy

The body of literature describing gene-expression changes through radiotherapy is highly heterogenous in terms of definition of the study cohort, timing of sampling and technologies. A combination of these factors of heterogeneity, small studies, their retrospective nature, and lack of validation means that conclusions of these studies should be interpreted with caution. Nonetheless, this body of literature provides material for hypothesis generation which will be useful for asking questions of a well-designed prospective study. The literature reviewed here indicates that neoadjuvant chemoradiotherapy can elicit immune changes within the tumour microenvironment involving various cell types and their associated responses. However, the mechanistic detail of the immune response to chemoradiotherapy remains elusive. Despite their widespread use, SCRT regimes are underrepresented in this literature. Future studies should seek larger cohorts stratified by treatment type, treatment response or consensus molecular subtype. Our current work aims to address these gaps through a prospective biospecimen retrieval protocol that will collect tumour biopsies from short-course and long-course treated rectal cancer patients for transcriptomic analysis with validation by immunohistochemistry at baseline, 2-week, 6-week and 12-week following the start of radiotherapy (Fig. 3).

## Data Availability

No new datasets were created or analysed for the purpose of this atricle.
